# Depth-Sensing-Based Algorithm for Chest Morphology Assessment in Children with Cerebral Palsy

**DOI:** 10.3390/s24175575

**Published:** 2024-08-28

**Authors:** Olivera Tomašević, Aleksandra Ivančić, Luka Mejić, Zorana Lužanin, Nikola Jorgovanović

**Affiliations:** 1 Faculty of Technical Sciences, University of Novi Sad, 21000 Novi Sad, Serbia; olivera.tomasevic@uns.ac.rs (O.T.); mejic@uns.ac.rs (L.M.); 2 Vukov Centar Os-Ossis, 21000 Novi Sad, Serbia; alexivancic@yahoo.com; 3 Faculty of Sciences, University of Novi Sad, 21000 Novi Sad, Serbia; zorana@dmi.uns.ac.rs

**Keywords:** depth-sensing, point-cloud data, wavelet transformation, patient morphology, chest mobility, children with cerebral palsy

## Abstract

This study introduced a depth-sensing-based approach with robust algorithms for tracking relative morphological changes in the chests of patients undergoing physical therapy. The problem that was addressed was the periodic change in morphological parameters induced by breathing, and since the recording was continuous, the parameters were extracted for the moments of maximum and minimum volumes of the chest (inspiration and expiration moments), and analyzed. The parameters were derived from morphological transverse cross-sections (CSs), which were extracted for the moments of maximal and minimal depth variations, and the reliability of the results was expressed through the coefficient of variation (CV) of the resulting curves. Across all subjects and levels of observed anatomy, the mean CV for CS depth values was smaller than 2%, and the mean CV of the CS area was smaller than 1%. To prove the reproducibility of measurements (extraction of morphological parameters), 10 subjects were recorded in two consecutive sessions with a short interval (2 weeks) where no changes in the monitored parameters were expected and statistical methods show that there was no statistically significant difference between the sessions, which confirms the reproducibility hypothesis. Additionally, based on the representative CSs for inspiration and expirations moments, chest mobility in quiet breathing was examined, and the statistical test showed no difference between the two sessions. The findings justify the proposed algorithm as a valuable tool for evaluating the impact of rehabilitation exercises on chest morphology.

## 1. Introduction

Cerebral palsy (CP) is a group of neurological disorders that affect movement and posture, resulting from non-progressive disturbances that occur in the developing fetal or infant brain [1]. While the primary manifestations of CP are related to motor function, chest wall deformities, such as pectus excavatum, and reduced chest wall mobility, can also be associated with this condition [2,3]. Their impact on respiratory function and overall well-being depends on the extent of the deformities and the individual’s specific motor and postural challenges [4]. Early detection, regular monitoring, and appropriate management can help mitigate the impact of the deformities on the patient’s respiratory and musculoskeletal physiology and optimize their health [5].

Some common treatments include physical therapy [6], bracing [7], and surgical interventions [8], and are performed from an early age in patients. Since it takes a comprehensive and individualized approach, physical therapy is considered to be a cornerstone of habilitative and rehabilitative care in the treatment of CP [9]. While the specific therapy stages may vary based on the individual’s condition [10], every approach requires treatment planning that is based on progress evaluation [11]. In this sense, for the patients undergoing treatment for chest deformities, reassessment of the morphological abnormalities is crucial [12]. In clinical practice, treatments for chest wall deformities and related decreased respiratory function are typically assessed through measurement tools such as measurement tapes and calipers [13,14] and cross-sectional imaging techniques like CT and MRI [15,16].

Namely, cross-sectional imaging techniques are typically used for chest deformity assessment. They can reveal abnormalities in the bony structures and also allow for precise quantification of deformity level, and a degree of correction after surgical intervention [16,17]. Since they offer detailed anatomical information, they have a great evaluation potential. However, their cost, availability, lack of real-time assessment, and most importantly, radiation exposure, omits them from routine assessment procedures in physical rehabilitation [18].

While imaging techniques are typically used for chest deformity assessment, the standard physical rehabilitation measurement tools, such as measurement tapes and calipers, allow the assessment of related, decreased respiratory function. In physical rehabilitation, respiratory function is typically quantified through chest expansion (CE) measure, derived in the thoracic excursion measurement process. Such papers focus on measuring the thoracic excursion as the difference between thoracic girth after maximal inspiration and at the end of maximal expiration [6,19,20,21,22,23,24].

In [19], chest expansion assessment was characterized as one of the simplest and most useful methods used to evaluate respiratory function. Using CE measurements, in [20], it was shown that chest mobility was decreased in spastic CP patients when compared to normal controls and concluded that early initiation of pulmonary rehabilitation seems reasonable in this patient group. Similarly, ref. [21] proved that in the waist expansion, children with spastic CP were significantly lower extensibility of the lung, compared to children with hemiplegic CP, and, thus, will require more intense monitoring regarding respiratory function in rehabilitation settings. On the other hand, ref. [6] assessed the therapy effect and proved that the CE increased significantly after using soft tissue therapy on the respiratory muscles.

Nonetheless, limitations about the subjectivity of measurements and their reproducibility have also been underscored [22,23,24]. These papers expressed the reliability of measurements through the inter-examiner intraclass correlation coefficient (ICC), based on which, in [23], it was concluded that CE taken at the level of the fourth intercostal space revealed poor to excellent reliability (ICC = 0.48–0.81) in the typically developing children group.

To overcome these limitations, there have been initiatives for circumference device implementation in the form of plethysmographs [25], optical sensors [26], capacitive sensors [27], wire-type linear encoders [28], and accelerometers [29], but what they encounter are limitations in wearability and portability, calibration and setup complexity, environmental sensitivity, contact or proximity requirements, limited measurement range, and limited depth information.

In recent years, depth-sensing technology has emerged as a promising tool for capturing and interpreting three-dimensional information about the environment [30], objects [31], and human interactions [32]. Compared to traditional distance measuring devices, depth sensing technology offers several advantages, including increased accuracy, objectivity, reproducibility, non-invasiveness, real-time feedback, and 3D imaging [33].

These also make them suitable for application in the healthcare environment. To name a few, it has been shown that depth sensing enables non-contact monitoring of vital signs [34] and capturing 3D anatomical data for diagnostics and surgical planning [35]. In the context of rehabilitation, the application boils down to patient monitoring, and specific applications are numerous. Namely, it is used for gait analysis [36], balance training [37], range of motion assessment [38], real-time feedback for physical therapy exercises [39], fall prevention [40], and home-based rehabilitation [41]. In these applications, depth-sensing allows the development of personalized rehabilitation plans, helps prevent injury, or allows therapists to monitor the progress of the patients remotely.

The aim of this work was to develop a simple and sufficiently precise objective method for tracking chest morphology changes in patients undergoing physical therapy. The proposed system is based on depth-sensing technology and robust signal processing algorithm designed to assess rehabilitation outcomes in children with cerebral palsy. The morphological parameters are extracted under the conditions of quiet breathing, and were also used to examine the change of chest mobility between the different sessions of the experiment.

This paper is organized as follows: first, depth-sensing methodology and the experimental setup are described, then, point cloud data processing and signal-processing algorithm applied to breath-induced variation in depth data are described, and in the end, the derivation of the final set of curves used to assess the chest morphology is described. Finally, the results are based on statistical tests that show how the condition of patients changes between the sessions of the experiment.

## 2. Proposed Methodology

### 2.1. Depth-Sensing Technology

Among the depth cameras available in the market, due to its versatile capabilities and high-quality depth information, the Intel RealSense D400 series (Attn: Corp. Quality, Intel Corporation, Santa Clara, CA, USA) has emerged as one of the leading options [42]. As representatives of active stereoscopy technology, D400 camera models encompass different depth ranges, resolutions, and field of view (FOV) options, providing flexibility for various use cases. With such characteristics, they are suitable for utilization in fields such as robotics and autonomous systems [43] and 3D scanning and modeling [44].

To minimize setup complexity and reduce motion artifacts, for the experiment, a wide field-of-view, global shutter, broad depth range camera, D435i (Attn: Corp. Quality, Intel Corporation, Santa Clara, CA, USA), was chosen. An overview of specifications is given in [45].

### 2.2. Subjects

Data acquisition was performed in the specialist clinic for physical medicine and rehabilitation “Vukov centar Os-Ossis” in Novi Sad, Serbia. Ten spastic cerebral palsy patients of the clinic (five males and five females, mean age 5 ± 2 years old, mean height 107 ± 8 cm, mean body mass 17 ± 3 kg) participated in the study. Their torso was inspected with a 3D camera. This study contains the results of the two acquisition sessions, performed with a time lapse of 2 weeks. The acquisition was performed with the consent of parents, and they signed the informed consent form for participation in the study.

### 2.3. Data Acquisition Set-Up

**Figure 1:**
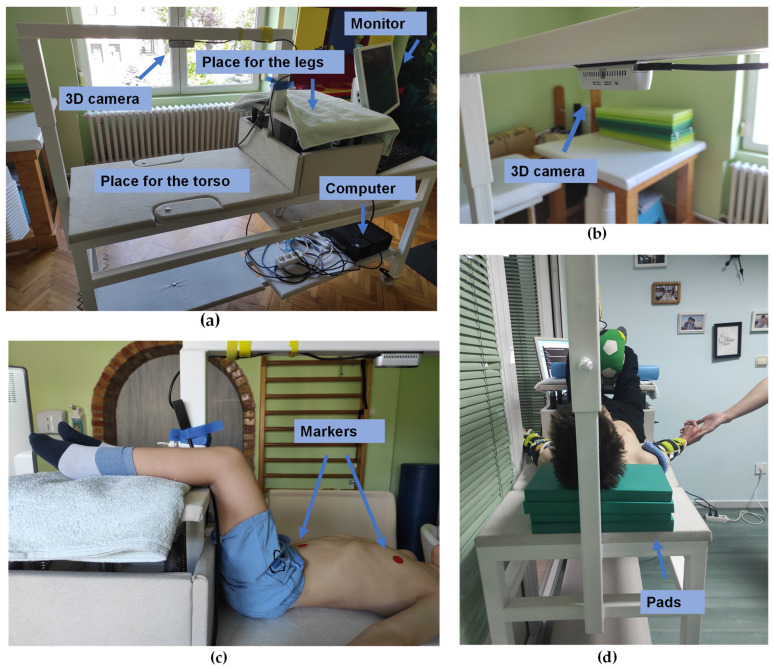
The experimental set-up: (**a**) the patient’s desk had a large surface for the patient to lay on it, and it also had a stand for the patient’s legs, and a bar above on which the 3D camera was fixed; (**b**) the camera was fixed to the bar with a black self-adhesive band; (**c**) the side view of the patient’s legs and torso—the position of the patient was always standardized—the lower legs were put on the stand and thighs were put closely to the leg support stand that was adjustable in height and adapted to each patient; (**d**) in some cases, the pads were placed under the head to ensure the optimal position of head and neck. Patients were always placed and positioned by the physical therapist.

The camera was placed above the patient’s desk (Figure 1) and fixed at approximately 530 cm. Patients were always placed in the same position, with their legs raised on the stand, so that there was as little opportunity for movement as possible, but in a position in which they could be completely relaxed. The acquisition was performed in two sessions, separated by a two-week interval. Each session comprised three trials, each lasting 60 s. Patients were instructed to be calm, and to breathe normally, i.e., without conscious or significant muscular effort.

Software for data acquisition was written in Matlab ver. R2021a (MathWorks, Natick, MA, USA) [46]. To set the camera acquisition parameters, the Intel’s SDK library [47] was used. The high accuracy setting and the frame rate of 30 fps were set. Data acquisition and processing were performed on a PC with Intel Core i5-9400 processor (Intel Corporation, Santa Clara, CA, USA) and 16 GB RAM.

### 2.4. The Algorithm

Since each session had fixed duration, every patient was initially represented with the same number of RGB-D images. The sequence of images was analyzed to isolate the change in the values of depth signal with time, in further text also referred to as a (breath-induced) depth variation signal. As this signal was considered as a representative of respiration process, it was perceived as a sequence of breathing curves, and it was searched for a dominant breathing pattern: a set of numerous breathing curves with similar properties.

When the breathing pattern was detected, depth information that temporally corresponded to such breathing was used to extract morphological cross-sections (CSs) for the moments of maximum (inspiration) and minimum (expiration) volumes of the chest.

**Figure 2:**
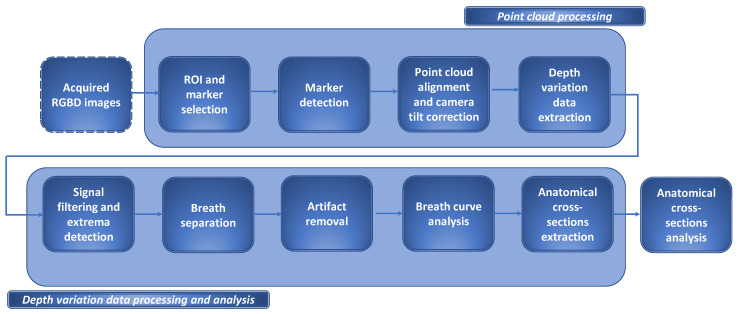
The proposed algorithm flow diagram. The figure illustrates all the phases of the algorithm—image acquisition, point cloud processing, depth variation data extraction and analysis, and morphological cross-section (CS) extraction and analysis. To allow the derivation of depth variation data, point cloud processing was applied to an acquired sequence of RGB-D images. Each image underwent a consistent chain of rigid transformations and was searched for the depth values on the previously determined coordinates of the grid of channels. Since depth variation data was perceived as represent of a respiratory signal, only the signal sections corresponding to the dominant breathing pattern were subsequently used for derivation of morphological CSs.

Thus, the proposed algorithm comprises depth (RGB-D) and depth variation data analysis and is depicted in Figure 2. The upper part of the figure illustrates the point cloud processing and the lower part illustrates the depth variation data analysis phase, in more detail.

### 2.5. Point Cloud Data Processing

The acquired RGB-D images had the structure of a colorized point cloud—every point was described by the point’s coordinate and color information. The color information served the visualization purposes, while the processing was primarily based on the value of the coordinates.

The point-cloud data processing was standardized in the following way: there were steps that were applied only to the first image of the observed trial and steps that were applied to each image of the trial. First image served for (1) region of interest (ROI) selection, based on which, every point cloud in the sequence would be cropped, and (2) marker points selection, based on which, every point cloud in the sequence would be rigidly transformed. Each next image was (3) subjected to automatic marker detection, (4) rigidly transformed so that it would be aligned with the principal axes in a specific way, (5) used to generate breath-induced depth variation data based on the location of marker points.

**Figure 3:**
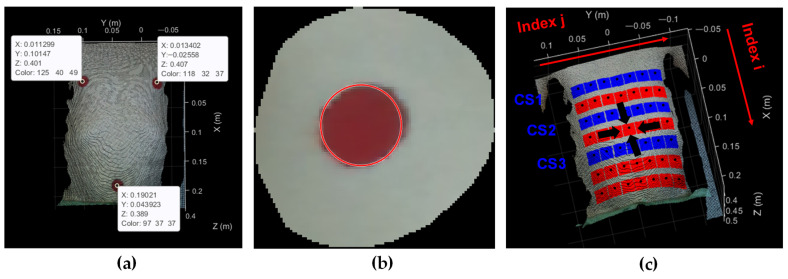
Cropped point cloud: (**a**) depicts the selection of marker points that was performed before rigid transformations; (**b**) depicts an example result of the marker detection. The detected marker is shown on an RGB image, which was obtained after the point cloud projection to the x–y plane; The dark red color represents the marker, while the light red color represents the marker boundary obtained after detection. (**c**) depicts the point cloud after rigid transformations—the resulting regions of interest (ROIs) are marked in red and blue, where blue represents the channels that were used for further analysis; (**c**) also illustrates the coordinate system for the enumeration of depth extraction channels—index i enumerates rows and index j enumerates columns of the grid of channels. Because of the rigid transformations, all the rows are perpendicular to the x axes, while all the columns are perpendicular to the y axes of the coordinate system. The ROI that is being pointed to in the image is the central channel of the grid, in the following text also referred to as the central location. The channels of interest for subsequent derivation of CSs are colored in blue.

The markers were made out of self-adhesive matte collage paper in a circular shape with a radius of 1 cm and were placed at the location of nipples and naval—Figure 3a. The marker detection process was iterative. The first frame of each trial was subjected to marker selection by the operator while all the other frames were subjected only to automatic marker detection. To further elaborate, the initial position of the marker that was determined by the operator was additionally corrected by applying the detection algorithm on the first frame of the sequence. The corrected location was used as initial location of the detection in the next frame, and the initial location narrowed the search area of the algorithm. Markers were detected using a circular Hough transform-based algorithm—it was performed in an HSV color space and applied to the saturation component of a 2D image obtained after the point cloud projection to the x–y plane. Figure 3b shows the detection result in the corresponding RGB image. In each frame, the central points in the marker area were calculated as points of interest and they were used for the generation of a grid of channels for the extraction of breath-induced depth variation signal. What followed for marker detection was rigid transformations of point clouds.

Since the camera’s x- and y-axis were largely aligned with the table’s longer and shorter peripheral edges, respectively, the point cloud was transformed in such a way that the line of the connection of markers set on the nipples matched the direction of the camera’s y-axis. This will later facilitate the extraction of transverse cross-sections (as points that share the same x-coordinate).

The rigid transformation also allowed the camera’s tilt correction. Considering that the camera’s z-axis was largely perpendicular to the table’s surface, after the tilt correction, all the points of the table in the resulting point cloud shared the same z-coordinate. Subsequently, points of interest were identified to extract depth variation data for further analysis.

All the points of interest were located in the intersection of the hypothetical lines parallel to the x and y principal axes that would be equidistantly distributed between the nipples and the naval, and the left and the right nipple, respectively. In this way, they formed a grid with 7 rows and 7 columns, whose intersection points are depicted in Figure 3c as black dots. Each point defined the region of interest (ROI) that was centered on it—the ROI was square-shaped with a vertex size of 2 cm. In Figure 3c, ROIs are represented in red and blue. Since these ROIs served for depth variation signal derivation, their layout was named as the grid of channels.

In order to reduce the amount of data for further processing while still allowing for the analysis of chest morphology, the channels of interest for derivation of CSs were chosen. They belonged to the 3 equidistantly distributed rows of the grid that completely or partially covered the chest region. They formed the grid 3 × 7, which is marked in blue in Figure 3c. Upon the analysis of those depth variation signals, morphological CSs were derived.

Although the channels of interest for further analysis belonged to the 3 × 7 subset of the grid of channels, in the first part of the study, the emphasis was placed on the central channel within the grid of channels, as the primary site for analyzing depth variation signal. It is being pointed to in Figure 3c. Due to its approximate equidistance from each marker (nipples and naval markers), it was hypothesized that it would exhibit greater resilience to imprecise marker selection or detection. Furthermore, since it aligned with abdominal breathing, it displayed more pronounced fluctuations in body volume and resulted in larger amplitudes of the depth variation signal, which enhanced the reliability of the corresponding depth variation data analysis. In the following text, this channel is also referred to as the central location.

### 2.6. Mathematical Representation of the Grid of Channels

If the grid of channels is represented as a matrix:

(1) $$X_{7\times 7}={\left[x_{ij}\right]}_{7\times 7},$$

where i are the rows and take values i=1, 2,…7, and j are the columns and take values j=1, 2,…7; then, the channels of interest belong to the 1st, 3rd, and 5th row of the matrix $X_{7\times 7}$, and will have the following elements:

(2) $$X_{3\times 7}=\left[\begin{array}{ccccccc} x_{11} & x_{12} & x_{13} & x_{14} & x_{15} & x_{16} & x_{17}\\ x_{31} & x_{32} & x_{33} & x_{34} & x_{35} & x_{36} & x_{37}\\ x_{51} & x_{52} & x_{53} & x_{54} & x_{55} & x_{56} & x_{57} \end{array}\right].$$

The indices of rows and columns are illustrated in Figure 3c. $x_{ij}$ will be the depth variation signal derived from the channel with (i,j) pair of coordinates.

### 2.7. Depth Variation Data Extraction

**Figure 4:**
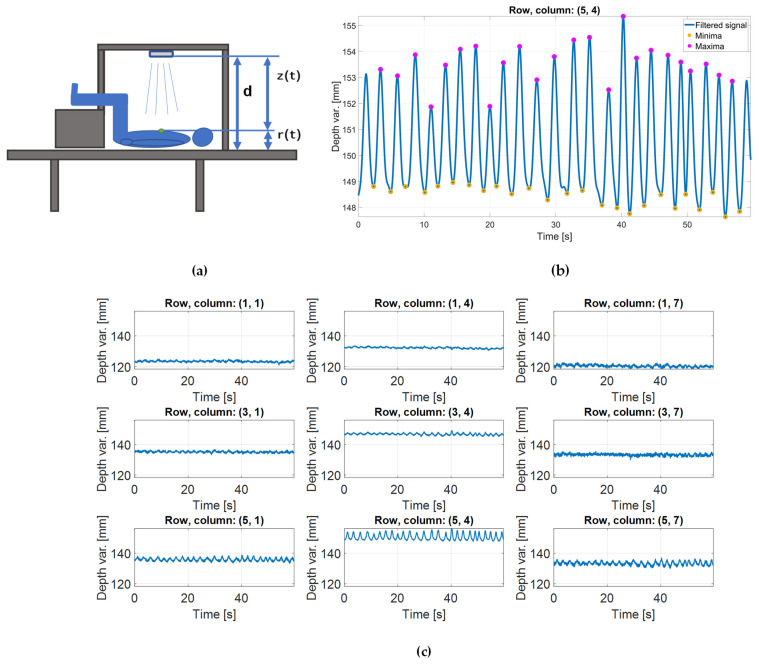
Derivation of depth variation signal: (**a**) illustration of variables used to calculate the depth variation signal, r(t), in a single point of interest. Green point is an observed single point; (**b**) the zoomed-in low pass filtered depth variation signal originating on one of the channels—(i, j) = (5, 4)—along with the detected extrema that were used for breath separation; (**c**) example of depth variation signals derived from 9 depth extraction channels that represent a subset of the channels of interest, a single trial.

Depth variation data were derived as a change in torso depth on previously defined areas of interest. Torso depth in a specified surface point was defined as the shortest distance to the table, and, therefore, calculated as a difference of the z-coordinate of the table (d), and z-coordinate of the surface point (z), respectively. In such a manner, the depth variation signal, $r\left(t\right)$, on a single point of interest was calculated as a change of torso depth with time:

(3) $$r\left(t\right)=d-z\left(t\right),$$

where d represents the camera-to-table distance, and $z\left(t\right)$ is a change of points z-coordinate with time. These variables are illustrated in Figure 4a. As one depth variation signal was calculated for the entire ROI that makes a single channel, the final expression had the form

(4) $$r\left(t\right)=\frac{1}{N}\sum_{i=1}^{N}\left(d-z_{i}\left(t\right)\right).$$

Here, $z_{i}\left(t\right)$ is a z-coordinate of an observed point belonging to the squared ROI that comprises of N points (depicted in the grid of channels in Figure 3c in red and blue). Such signals were calculated for every trial and each of the 21 channels of interest. Even though the final results are expressed for said 21 channels, for the sake of simplicity, most of the example results shown in this paper regard to a sparser grid of 3 × 3 channels that cover the chest region and belong to the central and two peripheral columns of the grid. Those signals belong to the following locations:

(5) $$X_{3\times 3}=\left[\begin{array}{ccc} x_{11} & x_{14} & x_{17}\\ x_{31} & x_{34} & x_{37}\\ x_{51} & x_{54} & x_{57} \end{array}\right].$$

To a certain extent, they illustrate the depth variation signals that were used for the morphological CSs extraction. An example of such signals is given in Figure 4c.

### 2.8. Depth Variation Data Analysis

Since it was considered that extracted depth variation signal was breath-induced, the assumption was that with the appropriate signal analysis, the repeatability of a certain breathing pattern could be detected, and a smaller portion of the signal representing artifacts could be removed.

Considering that the dominant breathing pattern was defined as a set of breathing curves, the next step was to perform breath separation. Similarly to the solution from [48], and assuming similarity with a respiratory signal, a breathing curve was extracted as a signal section extending between two consecutive minima of a depth variation trace. Since the acquired data were noisy, before extrema localization, the signals were smoothed with a 4th-order Butterworth low-pass filter with a cutoff frequency of 1 Hz.

On such smoothed signals, local extremes were identified as data samples larger than its two neighboring samples (local maxima) or data samples that temporally correspond to the local maxima of the inverted signal (local minima). Channels were not processed independently. In fact, indices of minimums and maximums detected on central locations were used for breath separation on every other channel as well. This ensured the same number of breaths in each channel.

The next phase included the removal of artifacts. Artifacts mainly originated from recording system deficiencies and movements of patients. Since these could be distinguished by larger amplitudes and/or sudden signal discontinuations, it was decided to analyze them through the lenses of continuous wavelet transform. These artifacts were searched for across all depth variation signals and all the signal sections that temporally corresponded to the onset of artifact in any of the channels were rejected. This method also removed a significant portion of outlying breath curves.

### 2.9. Continuous-Wavelet-Transform-Based Artifact Removal

Continuous wavelet transform is a mathematical tool that measures the similarity between a signal and an analyzing function. It is defined by the following expression [49]:

(6) $$W\left(a,b\right)=\int_{-\infty }^{\infty }x\left(t\right)\frac{1}{\sqrt{\left|a\right|}}\Psi \left(\frac{t-b}{a}\right)dt,$$

where parameter b translates the analyzing function Ψ along the signal $x\left(t\right)$, while parameter a variates the time scale of the analyzing function Ψ. The normalization factor provides that the wavelet energy is the same regardless of the parameter a value. One of the most widely used is the Morlet wavelet [50], and it is defined by the following expression:

(7) $$\Psi \left(t\right)=e^{-t^{2}}\cos\left(\pi \sqrt{\frac{2}{\ln\left(2\right)}}t\right).$$

**Figure 5:**
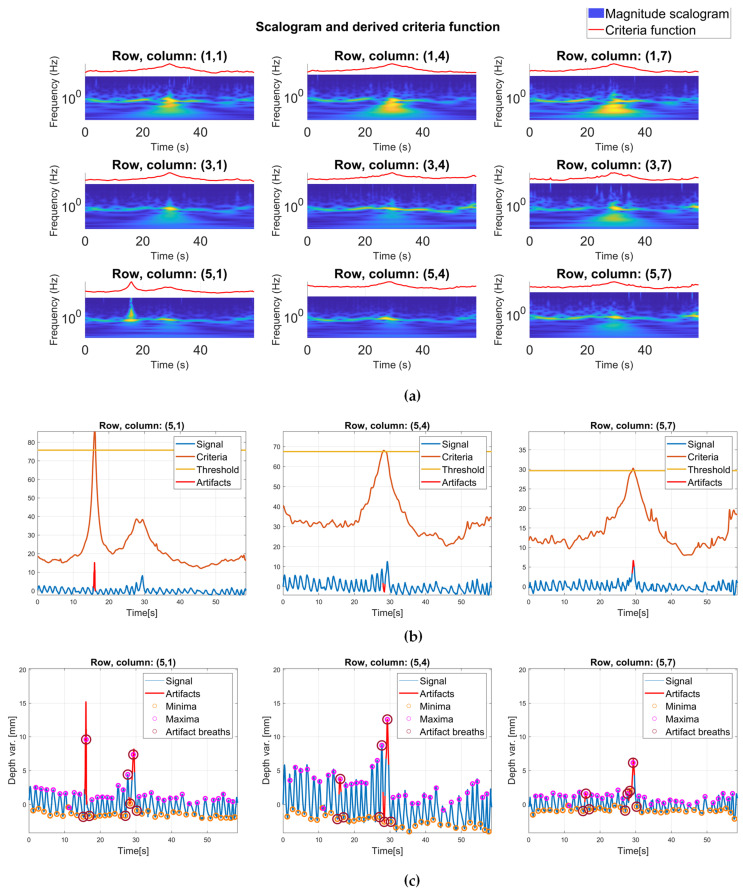
Artifact removal phase: (**a**) shows the magnitude scalogram of the wavelet transform of depth variation signals, and corresponding 1D power profiles (artifact removal criterion function), calculated for a single trial. A colormap is used to represent the magnitude of the wavelet coefficients—blue represents lower magnitude values, indicating weaker signal components at corresponding times and scales, while yellow represents higher magnitude values, and stronger signal components; (**b**) shows the criteria function more closely for the three channels of the 5th row—it can be observed that larger wavelet coefficients, as well as larger values of criteria function temporally correspond to the onset of artifact in depth variation signals. Threshold is a constant (yellow line); (**c**) shows artifacts detected on the same channels as in (**b**). The red full lines represent final artifacts: signal segments that temporally correspond to the onset of artifact on any of the channels. Red circles mark the extrema of breath curves that are contaminated with artifacts, and that will, thus, be omitted from further analysis; in this figure, all the signals are centered around zero for better visualization.

This wavelet was used for the observation of the change in the spectral content of depth variation signals with time. When observed on a scalogram, the artifacts were distinguished by the larger coefficient values. When summed across the scales, the scalogram generated a 1D power profile, in which the onset of the artifact could also be distinguished by larger signal values. This phenomenon detected on one trial is illustrated in Figure 5. Upon applying a threshold, the criterion functions on individual channels unveiled segments of the signal that contained artifacts.

The threshold applied to the criterion function was the 99 percentile of its values—it was adopted that the largest 1 percentile temporally corresponded to the onset of an artifact. Using this regularity, when identified, all the signal sections that temporally corresponded to the onset of an artifact on any of the channels were removed (as final artifacts). Figure 5a shows the scalogram of a trial that contains an artifact, and Figure 5b,c show corresponding criteria functions, and the results of artifact removal, respectively, for three channels. Since all the channels shared the same indices for breath separation, derived from extrema detection performed on the central location, after artifact removal, all the channels shared the same number of breaths retained for further analysis. Proposed artifact removal methods have proven to be effective and robust.

Each trial of the session was processed in the same way. Breath curves not considered artifacts were used in the breath curve analysis phase. Across all the 10 participants, in all three trials in total, in Session 1, the number of initial breaths was 94 ± 14, and the number of breaths retained after the artifact removal phase was 75 ± 10, and in Session 2, these numbers were 95 ± 13 and 77 ± 13, respectively. Further analysis combines all trials from the same session into a single group and examines the group as a whole.

### 2.10. Breath Curve Analysis

**Figure 6:**
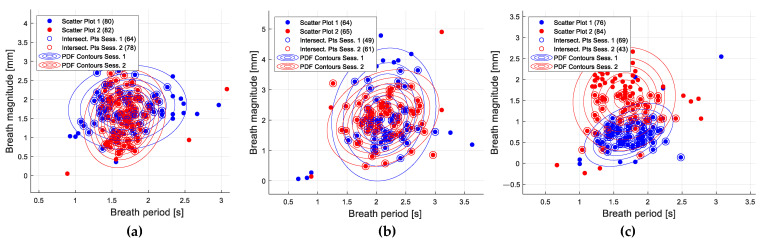
Breath curve analysis: the intersection of two Gaussian distributions with a probability of membership of at least 5% to each of the distributions gave a group of breaths that was used to generate morphological CSs. Graphs illustrate the projection of distributions to 2D Euclidean space, and the legend shows the number of breaths that belonged to both of the distributions. The results are depicted for three patients. In (**a**), there were 64 and 78 breaths in Session 1 and Session 2, in that order, retained for the next phase; in (**b**), there were 49 and 61; and in (**c**), there were 69 and 43 retained breaths, and 43 was the smallest number of breaths per patient that was retained for the next phase.

Since the aim was to compare the CSs between the two different sessions, a condition was set that the group of breaths based on which the CSs are drawn must be sufficiently similar. Breaths represented by breath width, i.e., period, and amplitude parameters were modeled by 3D Gaussian distributions. The intersection of two Gaussian distributions with a probability of membership of at least 5% to each of the distributions gave a group of breaths that was used to generate morphological CSs. Some typical examples of scatter plots depicting the characteristics of detected breaths as well as their similarities between the sessions are given in Figure 6. The smallest number of breaths per patient in any of the sessions retained for the next phase was 43—this case is illustrated in Figure 6c.

### 2.11. Point-Cloud Cross-Sections

After the removal of artifacts and outliers, the remaining breaths were used for derivation of the morphological CSs that corresponded to (a) moments of inspiration detected on the central location, and (b) moments of expiration detected on the central location.

In this way, every session was represented by a set of 2D curves that corresponded to the CSs of a point cloud. As depth variation signals from a 3 × 7 grid were analyzed, it was decided to derive CSs in the transverse, rather than in the sagittal morphological plane. Following the spatial arrangement of the channels explained in Section 2.5, the CSs themselves in the transverse plane were set in the direction in which the nipples were lying, and they were equidistantly distributed between row 1 and 5 of the grid. Although the CSs could have been extracted from the point cloud directly with a satisfying spatial resolution, it would be computationally more expensive than the extraction based on values of already existing depth variation signals. Thus, the chosen option was to derive the CSs based on values of depth variation signals.

For a specified row in a grid of channels, after connecting points of adjacent channels that temporally corresponded to the onset of inspiration, of each remaining breath detected on the central location, a set of curves was derived, which presented all the torso positions in inspiration phase (maxima CSs). Similarly, after connecting points of adjacent channels that temporally corresponded to the onset of expiration, the curves that presented all the expiration moments were generated (minima CSs). For both types (minima and maxima CSs), if it was extracted from the first row of the grid of channels (i=1), it was named first transverse cross-section (CS1); if it was extracted from the third row (i=3) second transverse cross-section (CS2); and from the fifth row (i=5) third transverse cross-section (CS3). These are also labeled in Figure 3c. In the new, cross-sectional representation of data, a CS is initially defined in 7 adjacent locations (j=1, 2, 3,…,7), and other values are obtained by linear interpolation.

**Figure 7:**
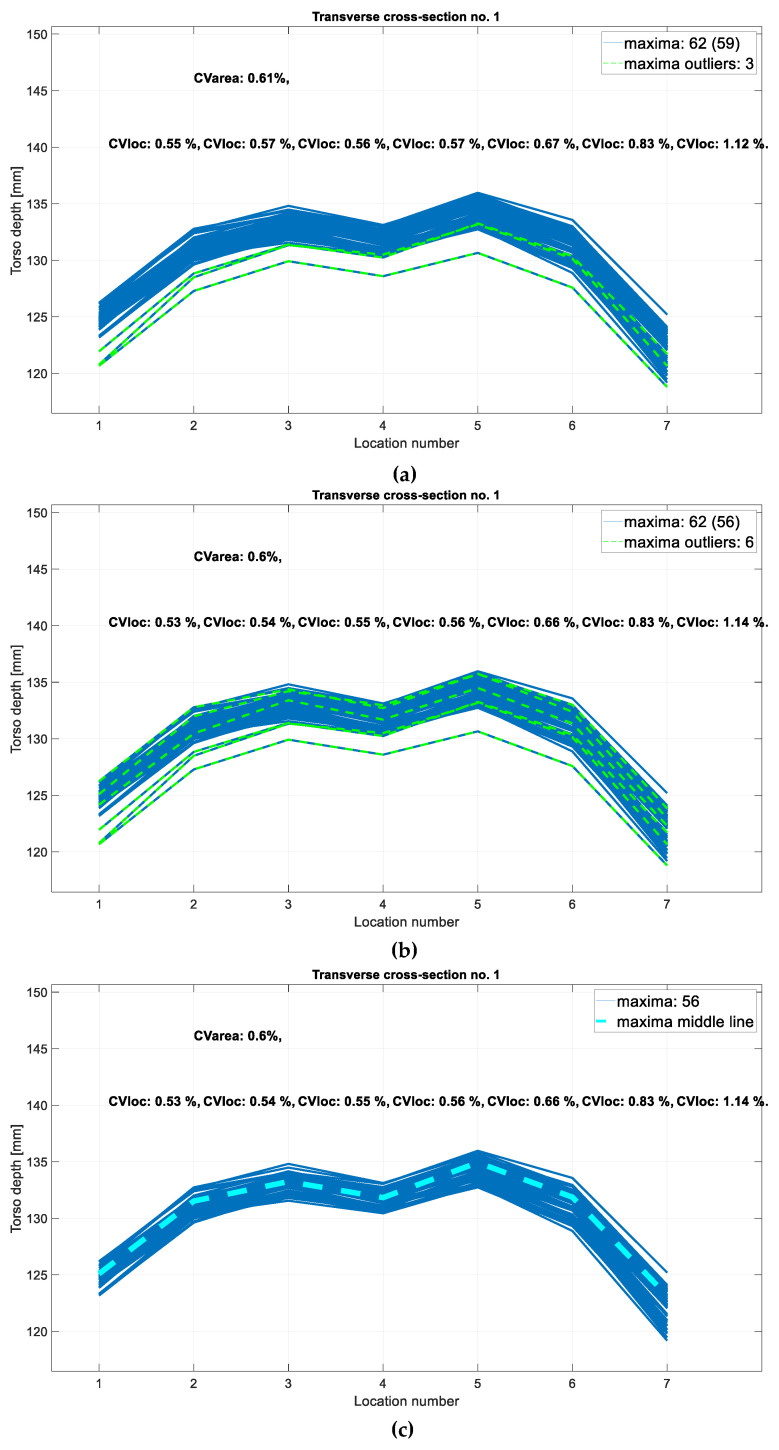
Cross-sectional representation of data illustrated for inspiration phase and in the level of CS1. CS was initially defined in 7 adjacent locations (j=1, 2, …7), and other values were linearly interpolated. Each graph represents a phase in CS processing, and all of them show the dispersion in the group of CSs—which decays when outliers are removed—the outliers were not included in the calculation; (**a**) shows initial outliers: a CS is an outlier if any of its data points is an outlier on an observed location j. If CS is an outlier, other CSs that are obtained for the same time sample are also considered outliers and removed, which is why the number of outlying cross-sections increases in (**b**); (**c**) depicts CS1 cross-section representative.

**Figure 8:**
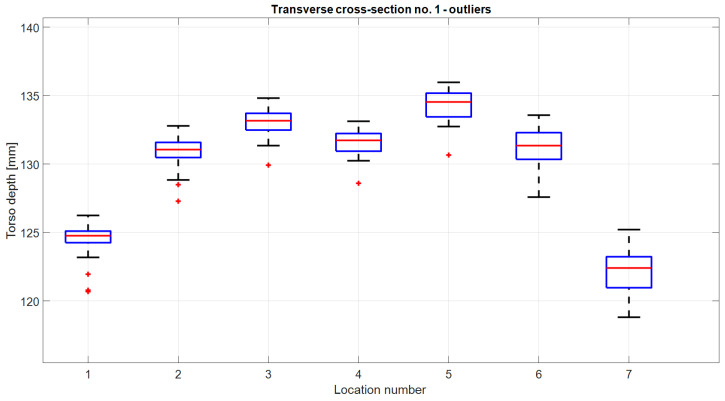
Boxplot of depth values of CSs acquired in different locations (j=1, 2, …, 7). Outliers were calculated for each location individually and are depicted as red plus signs. If an outlier was present in any location, the cross-section containing that point was also considered an outlier and removed. This figure illustrates how outlying CSs from Figure 7a were detected.

Figure 7 shows one example of inspiration CSs—CS1—and also illustrates outlier removal: if in any location, there was a torso depth value that was an outlier from the middle 50% of the depth data by more than 1.5 interquartile ranges (IQR), the entire CS was considered to be an outlier. These outlying CSs are marked in green in Figure 7a and explicitly shown on a boxplot of depth values in Figure 8. If CS was an outlier, other types of CSs that were obtained for the same time sample were also considered outliers and removed. That is why in the next phase of outlier removal, there were more detected outliers (Figure 7b). After outlier removal, the representative triplet of CSs (three CSs extracted for the same time sample) was calculated in the tridimensional feature space where every CS was represented by its area. The triplet whose areas formed a point that minimized the distance to other points in the feature space—a medoid—defined the representative CSs for the entire session. Representative CS for the first transverse cross-section is illustrated in Figure 7c. All the graphs in Figure 7 also contain the dispersion values in the group of CSs: dispersion is calculated as a coefficient of variation (CV) and calculated for each location along the CS and also as a CV of the CS area. This is also used in the next section to analyze the validity of the results.

After outlier removal, the number of CSs that was used for the derivation of representative CSs was larger than 30, in all the groups of CSs. In Session 1, in the group of inspiration CSs, there were 50 ± 6, while there were 65 ± 8 expiration CSs. In Session 2, there were 53 ± 14 CSs in the group of inspiration CSs and 64 ± 15 CSs in the group of expiration CSs.

## 3. Results

### 3.1. Analysis of the Dispersion of the CSs

One of the objectives of the study was to demonstrate the representativeness of the extracted cross-section curves, ensuring that the derived parameters effectively characterized the observed patient’s condition.

**Figure 9:**
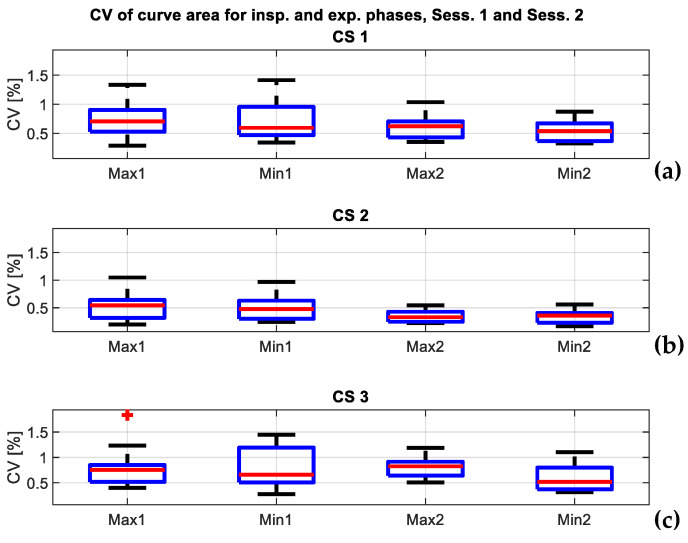
The coefficient of variation of areas under the CSs that belong to the same CS type. For each type of CS, the dispersion is calculated for maxima CSs (inspiration moments, Max), and minima CSs (expiration moments, Min). The left side of the graphs contains the results for Session 1 (Max1, Min1), while the right side contains the results for Session 2 (Max2, Min2); (**a**) shows the dispersion for the CS1; (**b**) shows the dispersion for CS2; (**c**) shows the dispersion for CS3; no value was greater than 2%, and the maximal value was 1.84% in the level of CS3 in Session 1. At the same time, this value is considered an outlier for the dispersion of maxima CSs in Session 1 in the CS3 level and is depicted as a red plus sign.

The dispersion of the curves in a session was expressed through the coefficient of variation (CV) of the estimated cross-section depth at the given location for the given type of CS (CS1, CS2, and CS3), and the coefficient of variation of the estimated area under the given type of CS (CS1, CS2, and CS3). The overall session results for all of the 21 channels are given in Table 1 (both sessions, minima CSs) and Table 2 (both sessions, maxima CSs). The dispersion of the curves was also expressed through the CV of the curve area, and shown in Table 3 (both sessions, minima and maxima CSs). As the values are represented for all of the ten patients, they are given in the form mean ± std. Dispersion in the curve area is also illustrated in the form of a boxplot in Figure 9.

In all three cases (dispersion on locations for expiration, dispersion on locations for inspiration, dispersion of CS area), both sessions shared similar values. For the expiration phase, the mean CV for locations was smaller than 1% in 19 out of 21 locations for Session 1, and smaller than 1% in all the 21 locations in Session 2. Greater mean values were 1.11 and 1.1% in the peripheral row and peripheral column of the observed part of the grid of channels (Table 1, Session 1). For the inspiration phase, mean CV was smaller than 1% in 17 out of 21 locations for Session 1 and in 18 out of 21 locations for Session 2. Greater mean values were 1, 1.06, 1.04, and 1.08 (Table 2, Session 1) in the peripheral columns and peripheral row and 1.03, 1.17, and 1.04 (Table 2, Session 2) in the peripheral row of the observed part of the grid of channels. The mean value of the CV of the CS area was smaller than 1% in all the CS types and in both sessions. The value per patient was smaller than 2% in all the CS types and in the both sessions, and the maximal dispersion was obtained at the level of CS3—1.84%, in Session 1.

### 3.2. Analysis of the Chest Mobility

After the analysis of individual session dispersion, the chest mobility under the conditions of quiet breathing was analyzed. It was quantified as a percentage change of the representative inspiration curve area relative to the representative expiration curve area. If a variable was introduced that stands for the area under the representative curve, *Acurve*, then the percentage change in the area under the representative maximum (i.e., inspiration CS), and representative minimum (i.e., expiration CS) would be:

(8) $$D_{sess}=\frac{A_{curve\;max}-A_{curve\;min}}{A_{curve\;min}}100\%,$$

where subscript max stands for the representative maximum, and min stands for the representative minimum.

**Figure 10:**
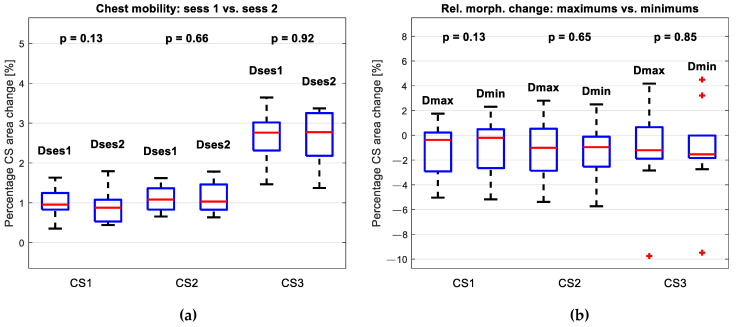
Results of statistical tests: (**a**) depicts the percentage change in the area of the inspiration representative curve relative to the expiration representative curve, a proposed indicator of chest The results showed that there was no statistically significant change in chest movement amount between the two sessions; (**b**) depicts change in morphology between the sessions; It was expressed as a percentage change in the area of representative inspiration curves (Dmax) and a percentage change in the area of representative expiration curves (Dmin); On the CS1 and C2 level, they were compared by a paired sample *t*-test, and on the CS3, they were compared by Wilcoxon signed rank test. Neither one of the tests showed a statistically significant difference between the groups. Outliers are represented as red plus signs, and are found in the CS3 level—one outlier for the change in representative inspiration curves, and three for the change in representative expiration curves. These were also included in the statistical test.

Figure 10a comparatively depicts the percentage difference between the representative CSs—maximum and minimum—for Session 1 and for Session 2, for each type of CSs (CS1, CS2, and CS3). These groups were compared. Since in all the groups, by the Shapiro-Wilkinson test [51], the data were normally distributed, the groups were subjected to paired sample *t*-test (with α = 0.05). The results are expressed numerically in Table 4.

Neither of the tests showed a statistically significant difference between the groups, which lead to the conclusion that the chest mobility did not change significantly in a two-week interval.

### 3.3. Analysis of the Relative Change in Morphology

In order to examine the relative change in morphology between the sessions, it was expressed as the percentage difference in area between the representative CSs of the two sessions. As the CSs were extracted for both inspiration and expiration moments of the depth signal, the change in morphology was expressed in two different ways: through percentage change in area of expiration representative CS, and percentage change in area of inspiration representative CS. If a variable was introduced that stands for the area under the representative curve, *Acurve*, then the percentage change between the sessions, for a given type of curve (either inspiration, i.e., maximum, or expiration, i.e., minimum), would be:

(9) $$D=\frac{A_{curve\;sess2}-A_{curve\;sess1}}{A_{curve\;sess1}}100\%,$$

where subscript sess1 stands for Session 1, and sess2 stands for Session 2.

Figure 10b comparatively depicts differences in representative maximums and differences in representative minimums for each type of CS (CS1, CS2, and CS3). The changes in the chest morphology based on the inspiration CSs were in the range −5 to 1.7% for CS1, −5.4 to 2.8% for CS2, and −9.8 to 4.2% for CS3, while the changes in the chest morphology based on the expiration CSs were in the range −5.2 to 2.3% for CS1, −5.7 to 2.5% for CS2, and −9.5 to 4.5% for CS3. Groups related to the same type of CS were compared. Since in the first two pairs of groups (related to CS1 and CS2), by the Shapiro–Wilkinson test, the data were normally distributed, the groups were subjected to paired sample *t*-test (with α = 0.05). In the third pair of groups, the first group was not normally distributed, so the groups were subjected to the Wilcoxon signed rank test (for paired observations). The results are expressed numerically in Table 5.

Neither one of the tests shows a statistically significant difference between the groups: different types of CSs—representative maximums and minimums—did not give different results. This leads to the conclusion that, if the breathing pattern is correctly detected, the CSs related to the maxima of the depth variation signal and CSs related to the minima of the depth variation signal can be interchangeably used to monitor the morphology of a patient.

### 3.4. Comparison of Morphological Parameters between the Sessions

**Figure 11:**
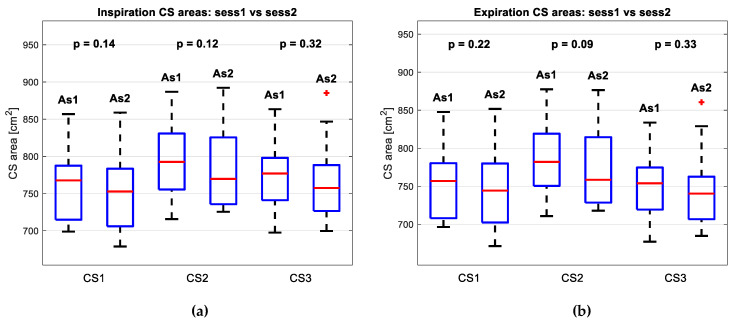
Results of statistical tests. Both figures depict the parameter area under the representative CS curve, comparatively depicted for Session 1 and Session 2; (**a**) shows the comparison of inspiration CSs, while (**b**) shows the comparison of expiration CSs; neither of the results indicate a statistically significant difference between the sessions; Red plus signs in the CS3 level of Session 2 represent the outliers. They were also included in the statistical tests.

As can be seen from Figure 10b, in individual cases, the area under the CS changed in both directions—it increased and decreased. However, additional statistical tests also compared the two sessions by the area parameter directly. Since all the groups were, by the Shapiro–Wilkinson test, normally distributed, the groups were subjected to paired sample *t*-test (with α = 0.05). The results are given in Figure 11a for maximum CSs and Figure 11b for minimum CSs. The results are expressed numerically in Table 6. Since neither of the results indicated a statistically significant difference between the sessions conducted only two weeks apart, the hypothesis of measurement reproducibility was confirmed.

## 4. Discussion

The study explored the applicability of a depth-sensing-based measurement system for the tracking of chest morphology changes in children with cerebral palsy. The new quantities and a novel method were introduced that yield parameters sufficiently indicative to track the same aspects as traditional methods, though not with identical metrics. While standard methods exist, this new approach aims to ease the process for both patients and doctors by providing alternative parameters that deliver the same essential information.

All the results were based on the extraction of morphological CSs that presented moments of inspiration and expiration under the conditions of quiet breathing. The reliability of the results was expressed through the similarity of extracted curves. Observed across all the patients, and both sessions, the mean CV of the assessed depth was smaller than 2% in all, and smaller than 1% in most of the locations, and this was observed in both sessions (Table 1 and Table 2). Similarly, the mean CV in the CS area was smaller than 1% in all the CS types and this was observed in both sessions (Table 3). Additionally, observed across all the patients, and both sessions, the CV in the CS area was smaller than 2% in all the CS types (Figure 9). Since the variation of curves was similar in the first and second sessions, it can be concluded that the method gave repeatable and reliable results.

In both sessions, the smaller values of CV were obtained in the internal locations, i.e., central to the observed part of the grid of channels, while peripheral locations had greater variations. One possible cause is the position of a child that, even though it was standardized, can never be entirely identical between the sessions. In support of that is the fact that children were sometimes additionally positioned between the two trials of the same session. The positioning of the chest in relation to the table might be better controlled if an anatomical bed was used, as it could minimize variations in posture and support, leading to more reliable and accurate measurements.

Since the results were dependent on the success of the automatic marker detection algorithm, it can also be concluded that the presented detection algorithm had the expected performance for all the three locations (nipples and naval markers). The detection algorithm can be described as a frame-by-frame iterative marker detection process with operator-guided initialization. The markers were automatically detected in every frame, however, the initial position in the first frame was selected by the operator. The detection algorithm corrected the position of marker, which was used as the initial estimate for the next frame. The recursive nature of the algorithm significantly reduced the search area for the marker detection, and ensured accurate marker detection without the need for recalibration for different heights of the patients.

To minimize the influence of gastrointestinal filling on the marker’s position and, consequently, on the extracted depth values, each location of interest dependent on the marker location was defined as a squared area with sides measuring 2 cm each. This area served to suppress minor positional shifts by averaging any variations within its boundaries, ensuring more consistent measurements. It is important to note that the acquisitions took place at the same time of the day, for each session; however, in future research, it should also be ensured that patients refrain from consuming food and liquids for a certain period before the recording, and they should be given the opportunity to use the toilet beforehand.

The results show that the chest mobility in a respiratory cycle did not change significantly between the two sessions (Figure 10a). Given the short time interval of only two weeks, this outcome was anticipated. The consistent results suggest that the proposed system effectively detected the morphological changes induced by breathing and that the algorithm performed well in identifying breathing patterns. This stability implies that any future changes in chest mobility may be more apparent with extended therapy. However, this potential for change should be further validated in a suitable patient group to confirm the system’s effectiveness with different therapeutic approaches.

The proposed morphology assessment algorithm showed that when the differences between the sessions were analyzed, the representative CSs extracted for the expiration and inspiration moments did not show statistically significant differences (Figure 10b). Thus, it can be concluded that if the breathing pattern is correctly detected, maximums and minimums of the breathing trace can be interchangeably used to monitor the morphology of a patient.

The median percentage differences in morphology between the sessions were approximately 0% at the CS1 level, −1% at the CS2 level, and −1.5% at the CS3 level (Figure 10b). The greatest change in morphology was observed for one patient in the CS3 level, amounting to −9.8% based on maxima CSs, and −9.5% based on minima CSs (an outlier in Figure 10b). Since this level covered the abdominal region, the differences of this kind could have been expected. Since the variation in CSs for this patient was smaller than 1% in every CS type, this observation was included in the groups for statistical tests.

Finally, a statistical test compared sessions 1 and 2 based on the area under the curve of the representative CSs. For both the inspiration and expiration CSs, the results show no statistically significant difference between the groups. Given the short two-week interval between measurements, this aligns with the hypothesis of measurement reproducibility.

## 5. Conclusions

The research addressed critical shortcomings in the chest morphology assessment for cerebral palsy patients: subjectivity, static measurements, and limited functional insights. The system is proposed, which is based on depth-sensing technology, robust signal processing algorithms, and morphological CS analysis. In the analysis of the morphological CSs belonging to ten patients and two sessions of the experiment set only two weeks apart, the proposed setup provided repeatable and reliable results: the variability of derived CSs was not greater than 2% in either of the sessions, the change of chest mobility between the sessions was not statistically significantly different, and neither were the values of extracted morphological parameters. Based on the results, the algorithm demonstrated sufficient precision in detecting both breath-induced morphological changes and the breathing pattern. This precision was crucial for accurately calculating the morphological parameters that form the basis of the study’s findings.

It is justified to propose such a system as another means of evaluation of progress in physical therapy and it is likely that it would exhibit high measurement accuracy. However, since the focus of this study was the depth-sensing-based algorithm, the accuracy of the proposed system has not been examined. In the next phase, the system must undergo a validation phase, where it would be compared against another, already established measurement method on a larger number of patients.

Since the impact of additional therapy on morphological parameters and patient condition has not been investigated at this stage, this will also be addressed in future work, where the performance of the presented algorithm would be further investigated and potentially improved.

## Figures and Tables

**Table 1:** Dispersion of features for transverse cross-sections, expressed as coefficient of variation of estimated depth for each of 21 locations, mean and std value for ten patients, Session 1 and Session 2. Shown for expiration phase. Measures are expressed in percentages.

**Exp. Phase (Sess. 1)**	**j = 1**	**j = 2**	**j = 3**	**j = 4**	**j = 5**	**j = 6**	**j = 7**
CS1	0.99 (0.51)	0.82 (0.41)	0.73 (0.33)	0.74 (0.33)	0.74 (0.33)	0.82 (0.34)	1.1 (0.34)
CS2	0.71 (0.32)	0.55 (0.24)	0.56 (0.35)	0.61 (0.34)	0.52 (0.23)	0.63 (0.24)	0.86 (0.3)
CS3	0.71 (0.34)	0.7 (0.28)	0.98 (0.46)	1.11 (0.64)	0.99 (0.64)	0.82 (0.4)	0.9 (0.27)
**Exp. Phase (Sess. 2)**	**j = 1**	**j = 2**	**j = 3**	**j = 4**	**j = 5**	**j = 6**	**j = 7**
CS1	0.82 (0.29)	0.68 (0.17)	0.61 (0.17)	0.51 (0.19)	0.6 (0.28)	0.7 (0.28)	0.96 (0.34)
CS2	0.76 (0.13)	0.46 (0.1)	0.36 (0.15)	0.4 (0.2)	0.4 (0.19)	0.5 (0.13)	0.8 (0.23)
CS3	0.72 (0.21)	0.64 (0.22)	0.8 (0.35)	0.9 (0.42)	0.81 (0.37)	0.67 (0.21)	0.85 (0.33)

**Table 2:** Dispersion of features for transverse cross-sections, expressed as coefficient of variation of estimated depth for each of 21 locations, mean and std value for ten patients, Session 1 and Session 2. Shown for inspiration phase. Measures are expressed in percentages.

**Insp. Phase (Sess. 1)**	**j = 1**	**j = 2**	**j = 3**	**j = 4**	**j = 5**	**j = 6**	**j = 7**
CS1	1 (0.51)	0.86 (0.35)	0.75 (0.3)	0.68 (0.34)	0.71 (0.32)	0.79 (0.31)	1.08 (0.25)
CS2	0.7 (0.25)	0.54 (0.24)	0.56 (0.33)	0.65 (0.41)	0.61 (0.32)	0.63 (0.22)	0.91 (0.35)
CS3	0.84 (0.35)	0.77 (0.37)	0.92 (0.53)	1.06 (0.55)	1.04 (0.52)	0.84 (0.41)	0.98 (0.31)
**Insp. Phase (Sess. 2)**	**j = 1**	**j = 2**	**j = 3**	**j = 4**	**j = 5**	**j = 6**	**j = 7**
CS1	0.95 (0.29)	0.77 (0.2)	0.67 (0.19)	0.6 (0.21)	0.63 (0.23)	0.73 (0.25)	0.93 (0.31)
CS2	0.76 (0.17)	0.44 (0.11)	0.36 (0.16)	0.4 (0.1)	0.45 (0.18)	0.52 (0.14)	0.78 (0.19)
CS3	0.73 (0.2)	0.77 (0.23)	1.03 (0.26)	1.17 (0.27)	1.04 (0.28)	0.82 (0.2)	0.85 (0.25)

**Table 3:** Dispersion of features expressed as coefficient of variation of area under each CS type, mean and std for ten patients. Shown for expiration and inspiration phase. Measures are expressed in percentages.

	Exp. Phase (Session 1)	Exp. Phase (Session 2)	Insp. Phase (Session 1)	Insp. Phase (Session 2)
CS1	0.74 (0.35)	0.55 (0.19)	0.73 (0.32)	0.62 (0.21)
CS2	0.52 (0.25)	0.34 (0.13)	0.54 (0.27)	0.34 (0.11)
CS3	0.77 (0.41)	0.61 (0.29)	0.81 (0.44)	0.83 (0.21)

**Table 4:** Results of the statistical test that compared the chest mobility between the two different sessions. The chest mobility was calculated as a percentage change in the area under the representative CSs for inspiration and expiration phases. Neither of the tests showed a statistically significant change between the groups.

CS1	t(9) = 1.66, *p* = 0.13
CS2	t(9) = −0.46, *p* = 0.66
CS3	t(9) = 0.10, *p* = 0.92

**Table 5:** Results of the statistical test that compared the change in morphology based on the areas of the maximum representative CSs, and minimum representative CSs. Neither of the tests shows a statistically significant change between the groups.

CS1	t(9) = 1.68, *p* = 0.13
CS2	t(9) = −0.47, *p* = 0.65
CS3	W = 30, *p* = 0.85

**Table 6:** Results of the statistical test that compared the area of the representative CSs between the two different sessions. First column is for the inspiration CSs, second for expiration CSs. Neither of the tests show a statistically significant change between the groups.

	Inspiration CSs	Expiration CSs
CS1	t(9) = 1.64, *p* = 0.14	t(9) = 1.32, *p* = 0.22
CS2	t(9) = 1.74, *p* = 0.12	t(9) = 1.88, *p* = 0.09
CS3	t(9) = 1.05, *p* = 0.32	t(9) = 1.03, *p* = 0.33

## Data Availability

The data presented in this study are available on request from the corresponding author. The data are not publicly available due to privacy reasons.
